# The Efficacy of VAMMFT Compared to "Bogota Bag" in Achieving Sheath Closure Following Temporary Abdominal Closure at Index Laparotomy for Trauma

**DOI:** 10.1007/s00268-023-06898-6

**Published:** 2023-03-30

**Authors:** Pavalini Pillay, Michelle T. D. Smith, John L. Bruce, Damian L. Clarke, Wanda Bekker

**Affiliations:** 1 Department of Surgery, Greys Hospital, Pietermaritzburg, South Africa; 2 School of Clinical Medicine, College of Health Sciences, University of KwaZulu-Natal, Durban, South Africa

## Abstract

**Introduction:**

The open abdomen (OA) is a necessary component of damage control surgery and closure is often challenging. Our aim was to review our ten-year experience with OA in trauma patients and to compare the success of a dual closure technique termed vacuum-assisted, mesh-mediated fascial traction (VAMMFT) to an exclusively Bogota Bag (BB) approach.

**Methods:**

A retrospective analysis was performed using the HEMR database from 2012 to 2022, comparing demographics, mechanism of injury, admission vitals and biochemistry between patients with BB and VAMMFT applications. Rate of secondary abdominal closure and complications were assessed in both groups. Logistic regression was used to find predictors of closure.

**Results:**

OA was required by 348 patients at index laparotomy. Of these, 133 (38.2%) were managed with VAMMFT and 215 (61.8%) exclusively with a BB. There were no statistical differences between the BB and VAMMFT groups in terms of demographics, injuries, admission vitals and biochemistry. The VAMMFT group achieved a closure rate of 73% compared to 54.9% in the BB group (OR of 2.2 [1.4–3.7]). There was no significant difference in fistulation rate between the two groups (*p* = 0.103). Length of hospital stay was 30 versus 17 days in the VAMMFT and BB groups, respectively (OR 1.41 [1.30–1.54]). There were no independent predictors of closure identified in the VAMMFT group. Older patients were less likely to achieve closure when BB was used (OR 0.97 [0.95–0.99]). VAMMFT failure was commonly due to lack of stock (39%) and protocol violations (33%).

**Conclusion:**

The VAMMFT approach to the OA is efficacious and safe. VAMMFT achieves a much higher rate of secondary closure than BB alone with a low rate of enteric fistula formation.

## Introduction

Following the widespread adoption of damage control surgery (DCS) and the increased awareness of intra-abdominal hypertension (IAH), across the globe, there has been an increased incidence of open abdomens (OA) [1–3]. Although the use of the OA may be lifesaving, it is itself associated with significant morbidity [1, 2]. An OA leaves the fascial edges widely separated and uses a temporary layer which contains and protects the underlying exposed viscera. This is referred to as temporary abdominal containment (TAC). TAC strategies should protect the viscera, allow drainage of abdominal fluid, and easy re-entry into the abdomen at subsequent laparotomy, be readily available and cost effective [1, 4, 5]. Definitive closure is achieved at a later stage. Delayed primary closure is the goal at second laparotomy. However, if this is not possible, the goal is to achieve secondary closure prior to discharge. If this is not achieved, a ventral hernia will result [1, 2]. Ventral hernias are morbid, impact negatively on quality of life and ability to work and require closure. This is a complex elective surgical procedure which is associated with significant morbidity. Considering this, major efforts have been directed at achieving delayed primary or secondary closure of all OA’s. There are three main approaches to achieving closure. There are devices which provide mechanical traction on the muscles of the abdominal wall, devices which exert a vacuum or negative pressure effect on the contents of the abdomen and, more recently approaches which combine both traction and negative pressure [3–5]. Our institution has used a combination of traction and negative pressure to achieve delayed closure for the last six years and the initial results have been published in the literature [6, 7]. Our approach has been termed the vacuum mesh-mediated fascial traction approach or VAMMFT. This study reviews our ten-year experience with OA and compares patients who were subjected to a VAMMFT approach with those who were managed exclusively with a BB approach.

## Definitions

This paper uses the following definitions: [6, 7]

- *Primary closure (PC):* Definitive closure at index laparotomy.
- *Open abdomen (OA):* Deliberate open abdomen requiring TAC.
- *Temporary Abdominal Containment (TAC):* A temporary layer which contains and protects the underlying exposed viscera.
- *Delayed primary fascial closure:* Fascial closure achieved at repeat laparotomy without the use of VAMMFT.
- *Secondary closure:* OA closed prior to discharge following the use of a TAC device.
- *Ventral hernia:* Primary abdominal fascial defect left open on discharge, covered with granulation tissue or skin graft, which will require fascial closure at a later stage.
- *Primary VAMMFT:* Application of VAMMFT at second laparotomy.
- *Secondary VAMMFT:* Application of VAMMFT following the second relaparotomy.

## Management of the OA by the Pietermaritzburg metropolitan trauma service (PMTS)

**Fig. 1:**
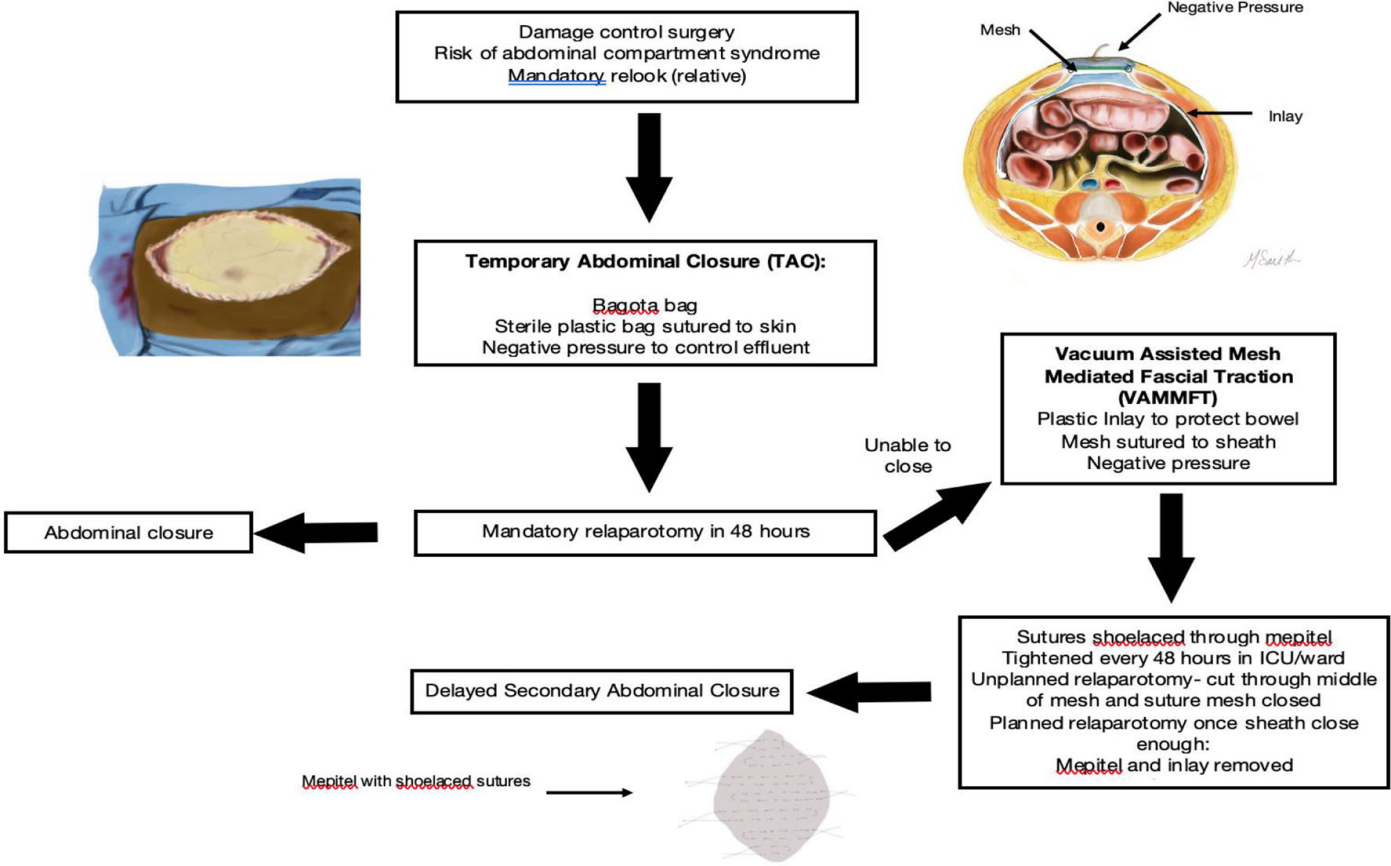
Protocol for management of the open abdomen in trauma patients

Prior to 2015, the OA was managed with a Bogota Bag (BB). This was applied at index laparotomy in patients whom primary closure at index laparotomy was deemed inappropriate. Once the patient’s physiology had stabilised, a repeat laparotomy was performed, and aggressive attempts were made to achieve delayed primary closure. If this was unsuccessful, a BB was reapplied, and the wound managed on its merits. If secondary closure could not be achieved, a split skin graft was applied to the granulated wound and the patient discharged with a ventral hernia. Ventral hernia closure was practiced as a delayed staged procedure once the patient had recovered. In 2015, the PMTS gradually introduced an approach to the OA called Vacuum-Assisted Mesh-Mediated Fascial Traction (VAMMFT). This approach is based on work originally popularised in the Scandinavian literature, modified and adapted to our local environment [2, 5]. VAMMFT was initially described by Rasilainen et al. and evaluated in our setting by Steenkamp et al. in 2017 [6, 8]. In our setting, all patients who undergo DCS or whose abdomen cannot be closed at the index laparotomy undergo a TAC in the form of a Bogota Bag (BB). These patients all return to the operating theatre as soon as physiology has been restored. At the first repeat laparotomy, if delayed primary abdominal closure cannot be achieved, a VAMMFT strategy is employed. This VAMMFT strategy uses a V.A.C Abdominal dressing system, (KCI, San Antonio, Texas USA) combined with a mesh-like sheet in the form of Mepitel^®^ Soft Silicone Wound Contact Layer (Molnlycke Health Care™ Dressings) which is sutured to the fascial edges with a continuous monofilament suture. A non-absorbable suture is shoe-laced through the mesh. A sponge is then placed over the mesh and the wound closed with an occlusive dressing. The dressing is perforated at the middle and connected to a continuous negative pressure suction device. The occlusive dressing and sponge are removed every 48 h, and the sutures over the mesh are serially tightened. This can be done in the ward in an aseptic manner under conscious sedation. If patients require repeat laparotomy, the mesh can be cut in the midline, and the KCI inlay removed to gain access to the abdomen. A new inlay can be placed, and the mesh sutured close if secondary closure cannot be achieved at that sitting. Once adequate fascial apposition has been achieved with serial tightening, patients can be taken back to theatre for definitive closure, which usually requires component separation and limited lateral release and a vacuum dressing over the skin, which is loosely opposed with interrupted sutures. A summary of the above management protocol is shown in Fig. 1. Patients who are discharged with ventral hernias are followed up at outpatient clinics by general surgery firms. These patients are usually booked for elective repair (with or without ostomy reversal as required) approximately nine months later. These ventral hernia repairs often involve extensive adhesiolysis, component separation of the abdominal wall and lateral release of the external oblique aponeurosis. Results of these repairs are beyond the scope of this study.

## Methodology

The Hybrid Electronic Medical Record System (HEMR) was established by the PMTS in 2012 and captures data on all trauma and surgical admissions. The HEMR was interrogated for all trauma patients admitted between 2015 and 2020, who underwent a laparotomy and who were left with an OA. Patients with grossly incomplete data, or who died prior to their first repeat laparotomy were excluded. Data meeting inclusion criteria were exported from HEMR as a single excel spreadsheet (Microsoft Excel 2018). Data were anonymised, except for the unique patient identifying number generated by HEMR. Data consisted of demographic data such as age, sex, mechanism of injury, vital signs on arrival, initial biochemical markers including serum lactate, pH, serum bicarbonate and base deficit. The shock index (SI) at presentation was recorded as well as the injury severity score (ISS). Operative details such as the nature of hollow viscus injuries were also recorded. Secondary abdominal closure following BB placement, primary VAMMFT and secondary VAMMFT were recorded, ventral hernias (VH), ICU admission and death. The reasons for VAMMFT failure were documented.

## Statistical analysis

The statistical method was that of a retrospective descriptive and inferential statistical analysis. Although a convenience sampling method was used, sample calculation was performed to ensure that the study would be adequately powered. A sample size of 134 patients with OA was required to estimate the characteristics of the cohort, in this case, closure of the abdomen to within 12% with 95% probability and using a non-informative estimate of 50%. Sample size was calculated using Stata V15.1 statistical software. Descriptive statistics were performed for the entire sample and subgroups. Patients who died with an open abdomen in hospital were excluded from this analysis (18). Continuous variables were described as medians and interquartile ranges due to non-normal distribution. Categorical variables were described as frequencies and percentages. Continuous variables were compared using the Wilcoxon test and categorical variables compared using the Chi-square test. Tukey’s transformation ladder was used to convert the dependent variable “length of stay” to a normal distribution. Univariate linear regression was used to examine relationships between method of TAC and length of stay. Logistic regression was used to examine relationships between binary outcomes and method of TAC. When *p* values were < 0.05, relationships were expressed as odds ratios and 95% confidence intervals. Data were analysed using R version 4.2.1 (The R Foundation for Statistical Computing). Patient consent was waived in accordance with class approval BCA 221/13.

## Results

**Fig. 2:**
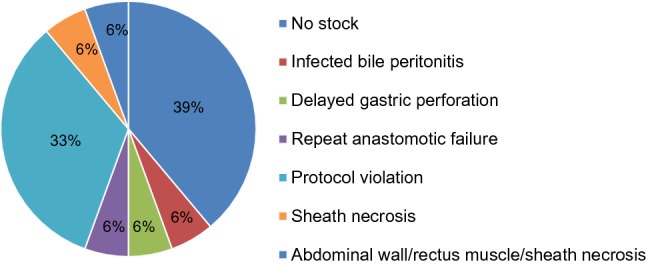
Pie chart categorising reasons for VAMMFT failure

Since 2015 a total of 348 patients required an OA as part of their surgical care. Of these, 133 (38.2%) were managed with a VAMMFT strategy and 215 (61.8%) with a Bogota Bag (BB) strategy. The median age was 31 years (IQR 25–40 years) with a male predominance of 85.6% (298 patients). The median ISS score was 14 (9–18) and 211 patients (60.6%) underwent DCS. The median SI was 0.92 (0.77–1.11) and the median serum lactate was 4.0 (2.0–6.0). Most patients sustained penetrating trauma (249; 71.6%). The most frequent hollow viscus injured was the small bowel (183; 52.6%) followed by the colon (143; 41.1%). No significant differences in demographics, admission biochemistry, admission vitals and type of injuries were observed between the two groups. Details may be seen in Table 1. The closure rate for the entire sample was 61.2% (202). Eighteen patients died prior to their repeat laparotomy and were excluded from analysis regarding closure. The overall mortality rate was 19.4% (67 patients). Abdominal closure prior to discharge was achieved in 73.0% (84) of patients with VAMMFT and 54.9% in the BB group. The odds of closure with a VAMMFT were 2.2 times that of a BB (95% CI 1.4 to 3.7). Conversely, there was an inverse relationship between VAMMFT application and VH (OR 0.45; 95% CI 0.27–0.73). There was no difference in rate of closure between primary and secondary VAMMFT, *p* = 0.686. Ten patients (3.0%) in the overall sample developed enterocutaneous fistulas. Our study found no significant association between TAC method and fistula rate. Patients with a VAMMFT application stayed longer in hospital than those with a BB (OR 1.4; 95% CI 1.30–1.54). Details can be seen in Table 2. Univariate logistic regression did not find any specific predictors of closure in the VAMMFT group. However, advancing age was inversely associated with abdominal closure in the BB group (OR 0.97; 95% CI 0.95–0.99). Details can be seen in Table 3. Each patient with VAMMFT failure was further audited for root cause. Figure 2 depicts the reasons attributed to failure. Most common was the lack of stock of the KCI inlay mesh, which was more prevalent in the earlier years included in this study. Protocol violation was second most common and refers to the VAMMFT not having been reapplied at repeat laparotomy without a valid reason. Patients with VAMMFT failure due to protocol violations were further analysed for details of the violation, with results listed in Table 4.

**Table 1:** Description of trauma patients undergoing TAC following index laparotomy

	All *N* = 348	VAMMFT *n* = 133 (38.2%)	Bogota bag *n* = 215 (61.8%)	*p*
Age years	31 (25–40)	31 (25–40)	31 (25–39)	0.833
Male	298 (85.6%)	117 (88%)	181 (84.2%)	0.328
ISS	14 (9–18)	16 (9–18)	13 (9–19)	0.919
DCS	211 (60.6%)	74 (55.6%)	137 (63.7%)	0.134
Shock index	0.92 (0.77–1.11)	0.93 (0.79–1.10)	0.91 (0.75–1.12)	0.659
s-Lactate	4.0 (2.0–6.0)	4.0 (2.0–6.0)	4.0 (2.0–6.0)	0.715
Small bowel	183 (52.6%)	71 (53.4%)	112 (52.1%)	0.815
Colonic injury	143 (41.1%)	53 (39.8%)	90 (41.9%)	0.711
Gastric	65 (18.7%)	22 (16.5%)	43 (20.0%)	0.421
Enteric all*	271 (77.9%)	99 (74.4%)	172 (80.0%)	0.224
Penetrating trauma	249 (71.6%)	98 (73.7%)	151 (70.2%)	0.488
Died	67 (19.4%)	26 (19.5%)	41(19.2%)	0.945

Continuous variables were described as medians and interquartile ranges. Categorical variables were described as frequencies and percentages. Alpha value set at 0.05

*Small bowel, colonic or gastric

**Table 2:** Association between method of TAC and in-hospital outcomes

	All *N* = 330	VAMMFT *n* = 115	Bogota *n* = 215	*p*	OR (95% CI)
Closed	202 (61.2%)	84 (73.0%)	118 (54.9%)	0.001	2.2 (1.4–3.7)*
VH	128 (38.8%)	31 (27.0%)	97 (45.1%)	0.001	0.45 (0.27–0.73)*
ECF	10 (3.0%)	6 (5.2%)	4 (1.9%)	0.103	
LOS	22 (12–35)	30 (21.5–43.5)	17 (10–29)	< 0.001	1.41(1.30–1.54)*

Patients who died with an open abdomen in hospital were excluded from this analysis (18). Continuous variables were described as medians and interquartile ranges. Categorical variables were described as frequencies and percentages. Tukey’s transformation ladder was used to convert the dependent variable “length of stay” to a normal distribution. Univariate linear regression was used to examine relationships between method of temporary abdominal closure and length of stay. Logistic regression was used to examine relationships between binary outcomes and method of TAC. When *p* values were < 0.05, relationships were expressed as odds ratios and 95% confidence intervals. (*) indicates statistical significance

**Table 3:** Predictors of closure by containment method group

Variable	VAMMFT	Bogota	
	*p*	*p*	95% CI
Age	0.136	0.013	0.97 (0.95–0.99)*
Male	0.518	0.381	
ISS	0.259	0.121	
DCS	0.088	0.532	
SI	0.847	0.409	
s-Lac	0.623	0.750	
Enteric	0.568	0.412	
Gastric	0.109	0.412	
Small bowel	0.305	0.077	
Colon	0.157	0.135	
Penetrating	0.321	0.126	
Primary vs. secondary VAMMFT	0.381	N/A	

Univariate logistic regression was performed for each subgroup to identify predictors of closure. There were no independent predictors of closure identified in the VAMMFT group. Age was inversely related to closure in the Bogota group. (*) indicates statistical significance

**Table 4:** Reasons for protocol violations

BB bag placed instead of VAMMFT (training issue)
Registrar did not call for help (training issue)
VAMMFT wrongly removed due to sepsis—not a contraindication
Consultant not trained to place VAMMFT
BB placed due to bowel oedema—not a contraindication
Inlay was cut too small (training issue)

## Discussion

While the OA may be a lifesaving strategy, it is associated with considerable morbidity. It is imperative to close the OA as rapidly and efficaciously as possible. These data have shown that VAMMFT (73%) is much more likely to achieve delayed closure than an exclusive BB (55%) approach. VAMMFT is also less likely to result in a ventral hernia. Although the length of hospital stay in the VAMMFT group was much longer than the BB group, this must be offset by the much higher rate of delayed closure and lower rate of ventral hernia. The rate of ECF was similar in both groups. VAMMFT does not promote enteric fistula. The cases of VAMMFT failure were frequently a direct result of protocol violation or in the initial period due to non-availability of the inlay device. This study has shown the inherent superiority of the VAMMFT approach over a BB approach. It has also demonstrated the safety of the VAMMFT approach. This is a single centre report and this makes it difficult to generalise these findings to other institutions. However, there is no reason why other institutions in South Africa with similar staffing and patient profiles as ours cannot implement a VAMMFT approach.

## Conclusion

The open abdomen is a necessary component of damage control surgery which is associated with high rates of ventral hernias. The VAMMFT approach to the OA is efficacious and safe. VAMMFT achieves a much higher rate of secondary closure than BB with a low rate of enteric fistula formation.
